# Investigating infectious outcomes in adult patients undergoing solid organ transplantation: A retrospective single-center experience, Paris, France

**DOI:** 10.1371/journal.pone.0291860

**Published:** 2023-10-05

**Authors:** Hassan Tarhini, Rami Waked, Mayda Rahi, Nihel Haddad, Richard Dorent, Christine Randoux, Vincent Bunel, Sylvie Lariven, Laurene Deconinck, Christophe Rioux, Yazdan Yazdanpanah, Veronique Joly, Jade Ghosn

**Affiliations:** 1 Service de Maladies Infectieuses et Tropicales, Hôpital Bichat Claude Bernard, AP-HP, Paris, France; 2 Division of Infectious Diseases, Maine Medical Center, Portland, ME, United States of America; 3 Service d’Hygiène Hospitalière, Pole Santé Publique, CHU Grenoble, La Tronche, France; 4 Service de Chirurgie Cardiaque, Hôpital Bichat Claude Bernard, AP-HP, Paris, France; 5 Service de Néphrologie, Hôpital Bichat Claude Bernard, AP-HP, Paris, France; 6 Service de Pneumologie, Hôpital Bichat Claude Bernard, AP-HP, Paris, France; 7 Université Paris Cité, Infection Modélisation Antimicrobial Evolution (IAME), Inserm UMR1137, Paris, France; Shiraz University of Medical Sciences, ISLAMIC REPUBLIC OF IRAN

## Abstract

**Objectives:**

This study described the demographic characteristics, clinical presentation, treatment, and outcomes of solid organ transplant recipients who were admitted to our center for infection. It also determined factors associated with a poor outcome, and compares early and late period infections.

**Methods:**

In this retrospective observational study, conducted at a tertiary care center in France between October 2017 and March 2019, infectious outcomes of patients with solid organ transplant where studied.

**Results:**

A total of 104 patients were included with 158 hospitalizations for infection. Among these 104 patients, 71 (68%) were men. The median age was 59 years old. The most common symptoms on admission were fever (66%) and chills (31%). Lower respiratory tract infections were the most common diagnosis (71/158 hospitalizations). Urinary tract infections were frequently seen in kidney transplant recipients (25/60 hospitalizations). One or more infectious agents were isolated for 113 hospitalizations (72%): 70 bacteria, 36 viruses and 10 fungi, with predominance of gram-negative bacilli (53 cases) of which 13 were multidrug-resistant. The most frequently used antibiotics were third generation cephalosporins (40 cases), followed by piperacillin-tazobactam (26 cases). We note that 25 infections (16%) occurred during the first 6 months (early post-transplant period). Patients admitted during the early post-transplant period were more often on immunosuppressive treatment with prednisone (25/25 VS 106/133) (p = 0.01), mycophenolic acid (22/25 VS 86/133) (p = 0.03), presented for an urinary tract infection (10/25 VS 25/133) (p = 0.04) or a bacterial infection (17/25 VS 53/133) (p = 0.01). Patients with later infection had more comorbidities (57/83 VS 9/21) (p = 0.03), cancer (19/83 VS 0/21) (p = 0.04) or were on treatment with everolimus (46/133 VS 0/25) (p = 0.001). During 31 hospitalizations (20%), patients presented with a serious infection requiring intensive care (n = 26; 16%) or leading to death (n = 7; 4%). Bacteremia, pulmonary and cardiac complications were the main risk factors associated with poor outcome.

**Conclusion:**

Infections pose a significant challenge in the care of solid organ transplant patients, particularly those with comorbidities and intensive immunosuppression. This underscores the crucial importance of continuous surveillance and epidemiologic monitoring within this patient population.

## Introduction

Solid-organ transplantation (SOT) is considered as the most effective treatment for selected patients with terminal organ failure [1]. While improvement in immunosuppressant agents’ efficacy has led to a decrease in the rate of organ rejection, it also increased the risk of opportunistic infections and morbidities in this population [2, 3]. The incidence and frequency of infections in this population varied and depended on various factors such as the type of transplanted organ, the level of immunosuppression, and the presence of comorbidities. Viral infections, including cytomegalovirus (CMV), Epstein-Barr virus (EBV), herpes simplex virus (HSV), varicella-zoster virus (VZV), and BK virus, are the most common infections in solid organ transplant patients [4]. Approximately 10% to 50% of solid organ transplant recipients develop CMV disease in the absence of prophylaxis [5]. Bacterial infections, including urinary tract infections, wound infection, and pneumonia, occur in up to 21% to 54% of transplant recipients [6, 7]. Parasitic and fungal infections such as toxoplasmosis, candidiasis and aspergillosis are less frequent and occur in 10% to 20% of patients [8]. Infections may increase mortality, development of malignancy and chronic allograft rejection [2]. It may lead to death complication in 21% to 63% of SOT recipients depending on transplanted organ [9]. The occurrence of infection in solid organ transplants is influenced by several factors: the type of organ transplanted, the level of immunosuppression, the use of antirejection therapy and the occurrence of complications during surgery [10].

Post-transplant infections can be divided into three time-periods: the immediate postoperative period (up to a month), the second phase (second to sixth months) and the late phase (beyond six months), each period having a predominant type of infection [2]. Most infections in the first month are nosocomial, donor derived or preexisting before transplantation such as latent tuberculosis, candidiasis, viral infection, bacteriemia or postsurgical infection. The second phase is typically characterized by the occurrence of opportunistic infections (Listeria, Nocardia, Mycobacterial infection) and immunomodulating viral infections such as CMV, EBV and BK virus infection or reactivation/recurrence of HBV or HCV [10]. After six months, patients may have a mildly increased risk for community-acquired infections due to sustained low doses of immunosuppressive treatment, chronic viral infection, and progressive organ diseases. Some patients may experience chronic rejection. They require an aggressive immunosuppressant treatment and are at high risk for opportunistic and nosocomial infections [6]. Several preventive strategies have demonstrated their efficacy: vaccination, prophylaxis and preemptive therapy [11]. The epidemiology of infections following organ transplantation has changed drastically since the implementation of prophylaxis protocols, better diagnostic tools and the increased potency of immunosuppressants and antirejection agents [12–17].

Knowing the microbiological epidemiology is crucial to properly treat such infections. In the present study, the primary endpoint was to describe the epidemiology of infections in immunocompromised solid organ transplant patients admitted to the infectious diseases department during an 18 month-period. In addition to that, secondary endpoints were investigated: analyzing the risk factors associated with early and late infections post transplantation, the association between transplantation types and specific infections, the risk factors associated with ICU admission or death.

## Material and methods

### Study design and setting

In this single-center retrospective cohort study, we reviewed the medical records of solid-organ transplant patients admitted to the infectious diseases department at the Bichat Claude Bernard University Hospital, Paris, France, between October 2017 and March 2019. Only those admitted for an infectious etiology were included in this study. No pediatric patient was admitted to this department. Transplant patients admitted to other departments of the hospital were not analyzed.

This study was approved by the “Comité d’Ethique de la Recherche (CER) Paris Nord” (Institutional Review Board -IRB 00006477- of HUPNVS, Paris 7 University, AP-HP). All patients received oral information from the referring physician at the time of consultation and provided their oral informed consent for data collection (between February and August 2022).

### Data collection

We extracted the following anonymous data from Orbis (Electronic medical records): demographic variables, comorbidities, type of organ transplantation, immunosuppressive drugs, clinical presentation at admission including fever, chills and symptoms and type of infection at admission, isolated pathogens, antibiotic /antiviral /antifungal treatment used and its duration, patients’ outcome, intensive care unit (ICU) admission, and type of complications. Only de-identified /coded data were exported from Orbis for analysis. Data were inserted in a standardized form for statistical analysis. Each admission to the hospital was counted as a separate entity which allowed the inclusion of the same patient several times.

### Immunosuppressive treatment and antimicrobial prophylaxis

Immunosuppressive treatment and antimicrobial prophylaxis for transplant recipients in our centre are in accordance with the International and European guidelines [18–20]. Immunosuppressive regimen consisted of the use of two or three different immunosuppressants, typically a combination of prednisone, one calcineurin inhibitor, and/or one antimetabolite.

All patients at our center received prophylaxis for pneumocystis using trimethoprim-sulfamethoxazole (160/800 mg) three times a week. Patients with Toxoplasmosis donor positive/recipient negative (D+/R-) status were given daily prophylaxis of trimethoprim-sulfamethoxazole (160/800 mg). Lung transplant recipients received fungal prophylaxis using nebulized liposomal amphotericin-B (25mg) twice a week, oral triazole antifungal (mostly voriconazole), or a combination of both treatments. The duration of prophylaxis ranged from 6 to 12 months, depending on mold infection risk factors. Other transplant recipients received amphotericin B oral suspension three times daily for at least 1 month as prophylaxis for oropharyngeal and digestive candidiasis. Additionally, clinical monitoring for invasive aspergillosis was recommended during the first month for all patients. A single dose of ivermectin 200 μg/Kg was administered to all patients before transplantation. Patients at high risk of CMV infection (D+/R-, D-/R+ or D+/R+) received prophylaxis with valganciclovir for 6 to 12 months following transplantation. All transplant patients were immunized with the influenza, pneumococcal, hepatitis B, and tetanus-diphtheria vaccines before transplantation.

### Definition of variables

Infections were defined and categorized according to the French Infectious Diseases Society recommendations [21]. Patients were classified as having one or several of the following infectious syndromes based on clinical and microbiological documentation in the patient’s medical chart: respiratory, urinary, digestive, skin and soft tissue, bone, neurologic, cardiac, or documented bacteremia. Microorganisms isolated in vitro were tested for drug resistance and classified as multidrug resistant (MDR), extensively drug resistant (XDR) or pan drug resistant (PDR) according to the recommendations of the “international expert proposal for interim standard definitions for acquired resistance” [22]. When viral reactivation and fungal infection were suspected based on clinical symptoms, diagnosis was performed using culture from respiratory secretions or body fluids, Polymerase Chain Reaction (PCR), respiratory multiplex PCR and serological tests. In patients without any confirmed microbiological cultures, a combination of the clinical and radiological results was used to categorize the infectious syndrome. Infections were classified as early or late, according to the post-transplant period (6 months period cut off with < 6 considered early). Nineteen different comorbid conditions were assessed according to the Charlson index [23]. The duration of hospitalization was calculated from the day of admission to the day of discharge. The time of observation was calculated from the day of transplantation to the day of hospital admission regardless of whether the patient was admitted more than once. Every hospitalization was counted as a unit of measure.

### Statistical analysis

Statistical analysis was performed using SPSS software. The normally distributed variables were expressed as means (±standard deviation) and compared with Student t-test. When the normal distribution condition was not respected, data were presented as medians (interquartile range), and compared with Mann-Whitney U Test. Categorical variables were expressed as numbers and percentages, compared using χ2 test with considering p values of 0.05 or less as statistically significant.

Then, we used logistic regression to identify risk factors associated with early and late period infection, death and ICU admission. Statistically significant factors as well as factors with a p-value between 0.05 and 0.25 were included in the binary logistic regression model to determine the independent predictive factors. The adjusted Odds Ratios (aOR) were also determined, Confidence intervals at 95% (95%CI) were also determined. Then p values of 0.05 or less were considered statistically significant.

## Results

### Patients’ characteristics

**Fig 1:**
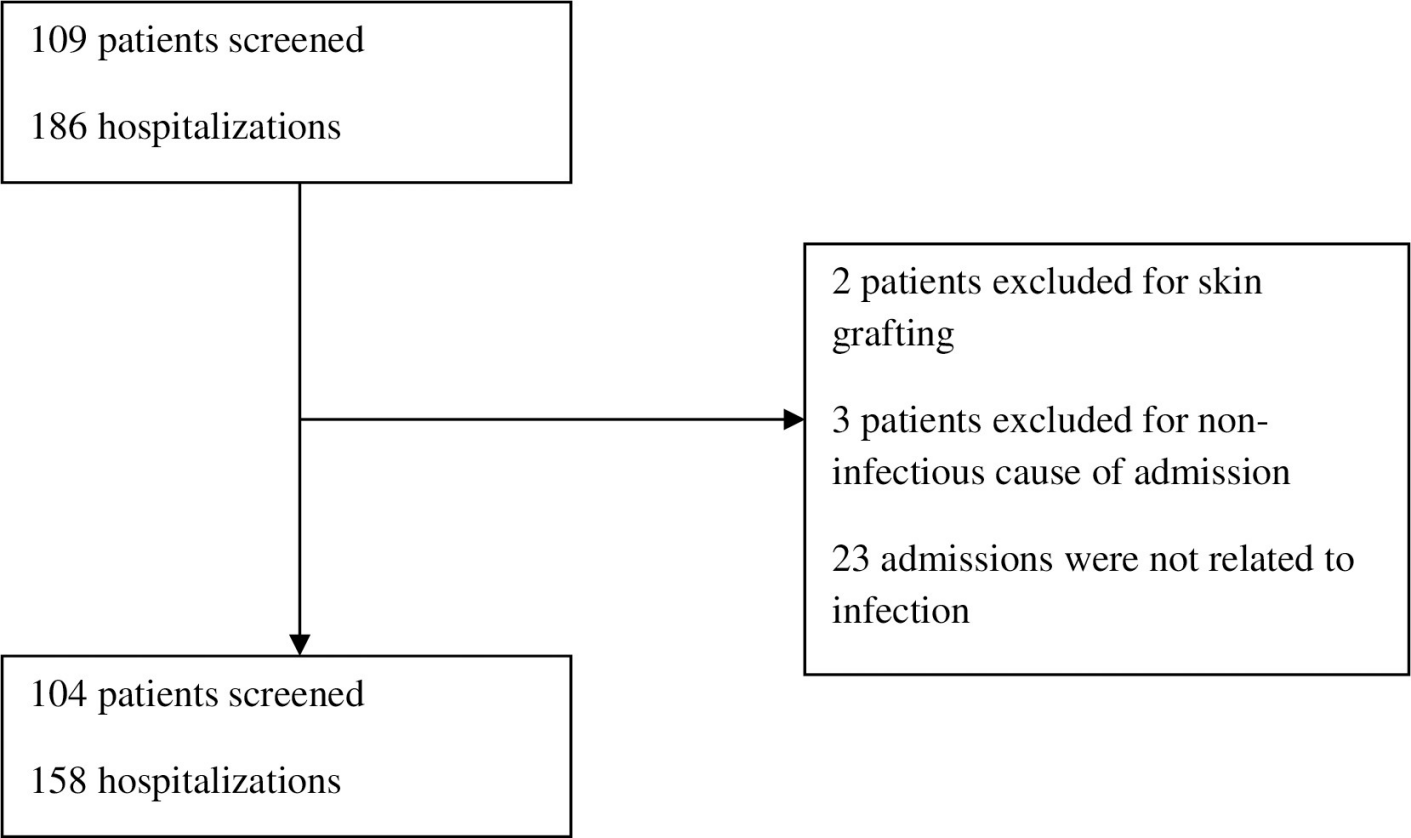
Flow chart of the study. From a total of 109 transplant patients, 104 patients presenting with infection were included. From these patients, 158 hospitalizations were caused by an infectious complaint.

A total of 109 solid organ transplanted patients were admitted to the infectious disease department during the study period with 186 distinct hospitalizations. Two patients were excluded for skin grafting, three transplanted patients were admitted for non-infection reasons and 26 admissions were not related to infectious complications. Infectious complaints resulted in 158 admissions accounting for 104 distinct patients (49 Kidney, 41 Heart, 9 Lung, 4 Liver, and 1 Kidney-Heart) (Fig 1). Median age was 59 years (range 25–81 years), 71/104 (68.3%) were men, 42/104 (40.3%) were diabetic and 66/104 (63.4%) had a Charlson comorbidity score ≥ 3. Nineteen (18.2%) patients had solid or hematological neoplasm and 10 (9.6%) patients were HIV positive. Twenty-six patients (25%) required more than one admission to the infectious department during the 18 months of the study with a maximum of 7 different hospitalizations for one patient. Most of these patients were cardiac transplant recipients. They were admitted for various infections. Some of them presented for a recurrence of their primary infection.

Among the 158 hospitalizations, there were 83 cases involving patients who had received heart transplants, 59 cases involving patients with kidney transplants, 9 cases involving patients with lung transplants, 6 cases involving patients with liver transplants, and 1 case involving a patient who had received both heart and kidney transplants. Demographic and clinical characteristics of patients are shown in Tables 1 and 2. One hundred thirty-three hospitalizations (84%) occurred after 6 months post transplantation. The median time between the date of transplantation and the date of infectious complication was 2 years with infections appearing earlier in cardiac transplants (1.7 years) compared to renal transplants, lung transplants or liver transplants (3, 3 and 12 years respectively). The median duration of hospitalization was 9 days.

**Table 1:** Demographic characteristics of the studied population and univariate analysis comparison between early and late period groups.

	Total study population	Early period infection	Late period infection	P-value
Number of patients	104	21	83	-
Age (years), mean (±SD)	59.7 ± 10	**55.0 ± 10**	60.6 ± 9	**0.01**
Male	71	14	57	0.77
Diabetes	42	5	37	0.09
Charlson score ≥ 3	66	9	**57**	**0.03**
HIV positive	10	4	6	0.24
Neoplasm	19	0	**19**	**0.02**
Transplantation type				
Kidney	49	7	42	0.13
Heart	41	12	29	0.20
Lung	9	2	7	0.99
Liver	4	0	4	0.42
Heart-Kidney	1	0	1	0.99

Early period: < 6 months between transplantation and infection

Late period: > 6 months between transplantation and infection

Values are expressed by numbers. Significant differences are highlighted in bold.

**Table 2:** Clinical characteristics of registered transplant admissions and univariate analysis comparison between early and late period groups.

	Total number of hospitalization	Early period infection	Late period infection	P-value
	158	25	133	-
Immunosuppressive drug used				
Prednisone	131	**25**	106	**0.01**
Cyclosporin	63	12	51	0.36
Tacrolimus	69	13	56	0.58
Mycophenolic acid	108	**22**	86	**0.03**
Everolimus	46	0	**46**	**0.001**
Azathioprine	18	1	17	0.35
Sirolimus	19	0	19	0.09
Immunosuppressor overdosage	26	7	19	0.16
Fever	105	15	90	0.60
Chills	49	12	37	0.07
White blood cell count (10^3^/dl), (mean ±SD)	7853 ± 4884	6329 ± 3819	8139 ± 5020	0.09
C-reactive protein, mg/L, median (IQR)	66.5 (0–487)	106 (0–331)	66 (0–487)	0.13
Site of infection				
Respiratory tract	71	13	58	0.57
Urinary tract	35	**10**	25	**0.04**
Intra-abdominal	18	0	18	0.10
Skin and subcutaneous	10	2	8	1
Osteo-articular	7	1	6	1
Blood stream	20	2	18	0.66
Nervous system	3	0	3	0.99
Cardiovascular	1	0	1	0.99
Isolated micro-organism				
Viral	36	6	30	1
Bacterial	70	**17**	53	**0.01**
Mycosis	10	2	8	1

Early period: < 6 months between transplantation and infection

Late period: > 6 months between transplantation and infection

Values are expressed by numbers. Significant differences are highlighted in bold.

**Fig 2:**
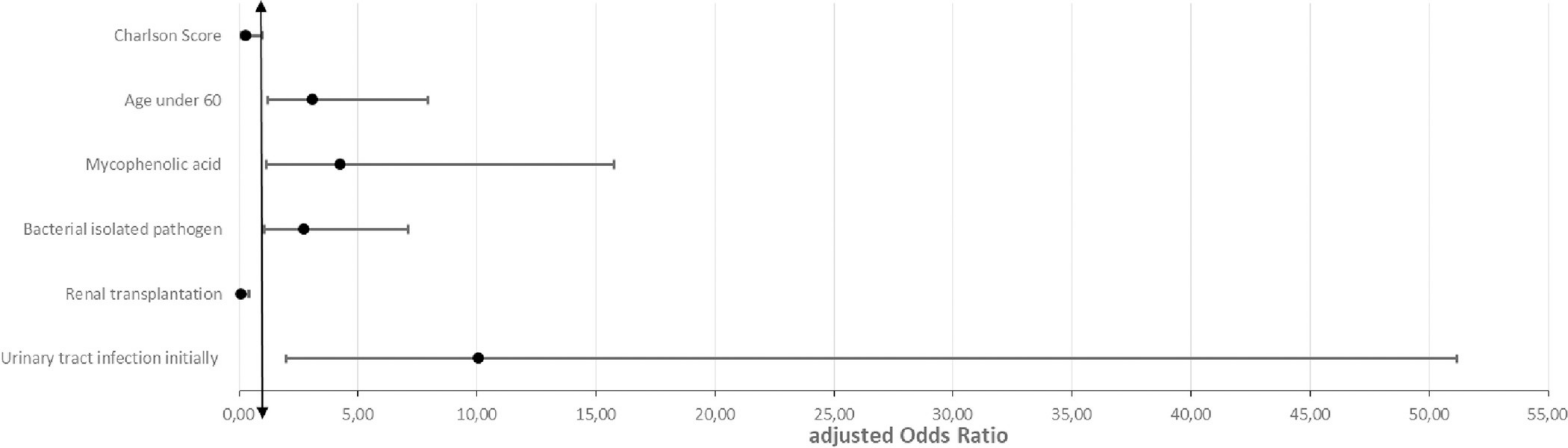
Regression plotting of risk factors for early period infection.

In the preliminary univariate analysis, patients admitted during the early period were younger, were more often on immunosuppressive treatment (prednisone (25/25 hospitalizations versus 106/133) (p = 0.01) or mycophenolic acid (22/25 VS 86/133) (p = 0.03)), presented with documented bacterial infection (17/25 VS 53/133) (p = 0.01) and most of them had urinary tract infection (10/25 VS 25/133) (p = 0.04). Patients admitted with late infection had higher Charlson scores (57/83 patients admitted in late period versus 9/21 admitted for the first time in early period) (p = 0.03, aOR 0.28), had a malignant neoplasm (19/83 VS 0/21) (p = 0.02) or were receiving everolimus (46/133 hospitalizations in late period VS 0/25 in early period) (p = 0.001). Subsequently, we performed logistic regression analysis. We found that patients admitted during the early period were younger (aOR for age under 60 = 3.11), on mycophenolic acid (aOR = 4.26), had bacterial infection (aOR = 2.75) or urinary tract infection (aOR = 10.08). Compared to early infections, patients with higher Charlson scores or renal transplantation had more frequent late infections (aOR 0.28 and 0.08 respectively). No significant difference was found between the two groups comparing other types of organ transplant or other sites of infection (Fig 2).

In 132 hospitalizations (83%), patients showed immunosuppressive therapy plasma levels within the expected range on admission. In the remaining 26 hospitalizations (17%), patients had elevated immunosuppressive plasma levels. Fever and chills were the most frequent admission symptoms accounting for 66.5% and 31% of hospitalizations, respectively. Other symptoms included cough (22%), dyspnea (15.2%), abdominal pain (14.4%) and dysuria (13.1%). The most common types of infection were respiratory followed by urinary tract infections (71/158 and 35/158 hospitalizations). Patients with renal transplantation had more urinary tract infections (UTI) compared to others (25/35 hospitalizations for UTI) (p < 0.001) (Table 3). No correlation was found in the other types of transplantations or for microbiological etiology (Table 4).

**Table 3:** Association between type of organ transplant and site of infection in the studied population.

	Kidney	Heart	Lung	Liver	Kidney and Heart	Total	p-value
Respiratory	18 (25.4)	44 (62.0)	4 (5.6)	4 (5.6)	1 (1.4)	71	0.078
Urinary tract	**25 (71.4)**	9 (25.7)	0 (0.0)	0 (0.0)	1 (2.9)	35	**<0.001**
Intra-abdominal	4 (22.2)	12 (66.7)	1 (5.6)	1 (5.6)	0 (0.0)	18	0.654
Cutaneous	3 (30.0)	5 (50.0)	2 (20.0)	0 (0.0)	0 (0.0)	10	0.248
Osteo articular	3 (42.9)	3 (42.9)	1 (14.3)	0 (0.0)	0 (0.0)	7	0.774
Bacteremia	8 (40.0)	11 (55.0)	0 (0.0)	1 (5.0)	0 (0.0)	20	0.808
Total	61	84	8	6	2	161	

**Table 4:** Association between type of organ transplant and microbiological etiology.

	Liver	Heart	Kidney	Lung	Kidney and Heart	Total	p-value
Bacterial^1^	1 (5.7%)	32 (45.7%)	30 (42.9%)	6 (8.6%)	1 (1.4%)	70	0.132
Viral^2^	3 (8.3%)	22 (61.1%)	10 (27.8%)	1 (2.8%)	0 (0.0%)	36	0.249
Mycosis^3^	0 (0.0%)	9 (90.0%)	0 (0.0%)	1 (10.0%)	0 (0.0%)	10	0.096

1: Most frequent bacteria: Escherichia coli, Klebsiella pneumoniae and Pseudomonas aeruginosa

2: Most frequent viruses: Rhinovirus, Influenza A and Coronavirus

3: Fungi: Aspergillus fumigatus

One or more pathogens were detected in 113 hospitalizations (71.5%). Bacterial, viral, or fungal infections were respectively documented in 70 (44%), 36 (22.8%), and 10 (6.3%) hospitalizations, while no microorganism was isolated in 45 cases (28.5%). The most frequent pathogens identified were Gram negative bacteria, with 18 cases of *Escherichia coli*, 12 cases of *Klebsiella pneumoniae* and 13 cases of *Pseudomonas aeruginosa*. Eight patients harbored an extended spectrum beta-lactamase producing bacteria, four harbored a carbapenemase producing *Enterobacteriaceae* and one harbored an MDR *Pseudomonas aeruginosa*. The most frequent gram-positive bacteria identified were *Enterococcus faecalis* (6 cases) followed by *Staphylococcus aureus* (4 cases). *Aspergillus fumigatus* was the most frequent fungi detected in cultures (5 cases). The most viral infections were respiratory such as Rhinovirus (10 cases), Influenza A (9 cases) and Coronavirus (5 cases). Reactivation of CMV led to the hospitalization of five instances involving three distinct patients. White blood count was higher in patients with bacterial infections (9500 G/L) compared to patients with viral (6500 G/L) or fungal infections (8500 G/L). C-reactive protein (CRP) was also higher in bacterial infections with an average of 122.5 mg/L. The most used radiologic test was the computed tomography (CT) scan (44%).

**Fig 3:**
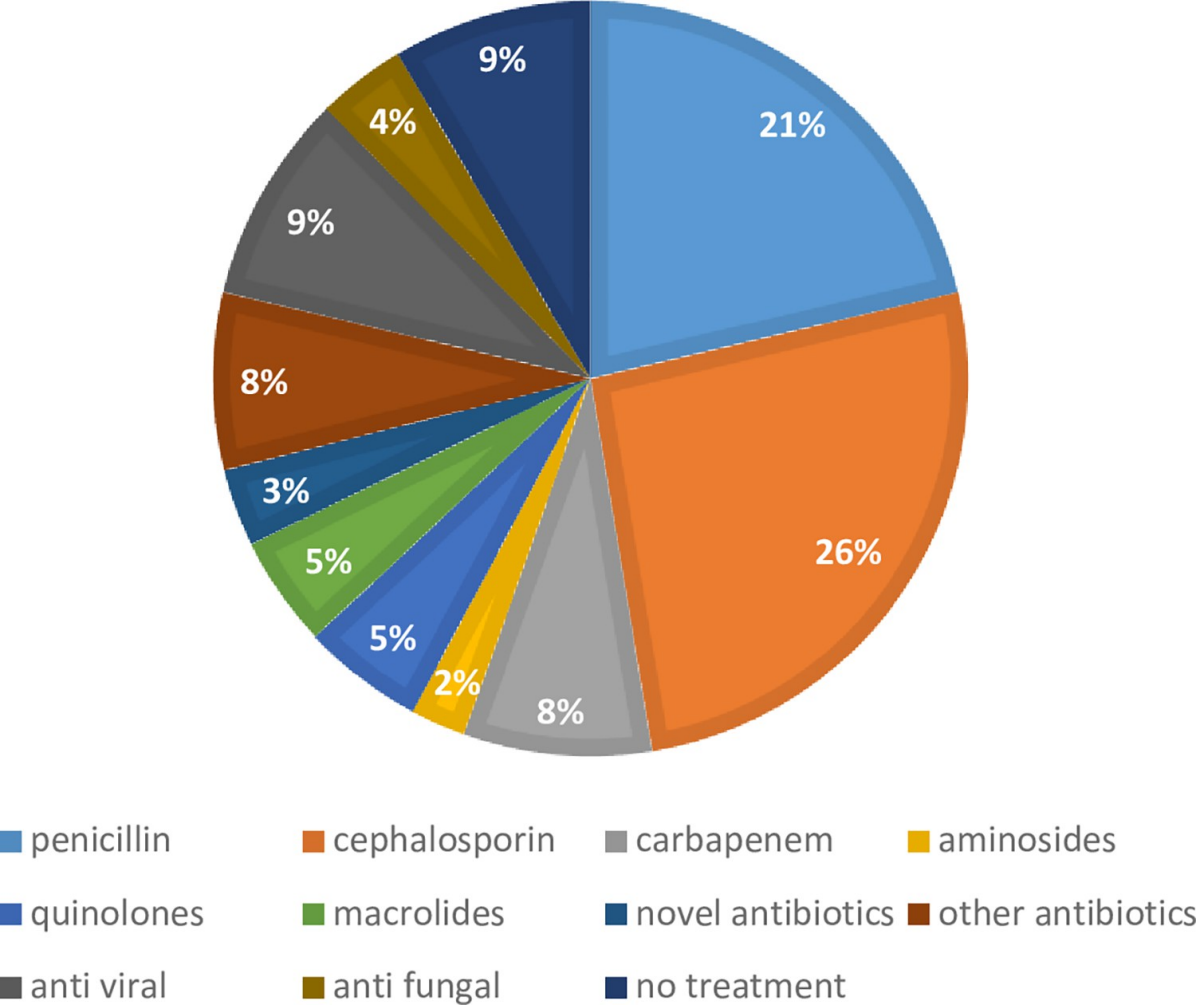
Treatment at admission. Novel antibiotics: ceftolozan /tazobactam, ceftazidime /avibactam.

Third generation cephalosporin was the most frequently used antibiotic among all admissions (40 cases for cefotaxime and ceftriaxone), followed by piperacillin-tazobactam (26 cases). In seven cases, patients were known to harbor an MDR pathogen and were started on Ceftolozane-Tazobactam (6 cases) or Ceftazidime-Avibactam (one case) (Fig 3). Voriconazole was the most used antifungal agent (5 cases) and Oseltamivir the most used antiviral agent (11 cases) (Fig 3). In 24 cases (15%), antimicrobial agents were either withheld initially or discontinued as no specific microbial agent necessitating treatment was identified.

A total of 31 cases (20%) needed ICU stay during their hospitalization. Amongst ICU admissions, piperacillin-tazobactam was the most frequently used antibiotic (8 cases) followed by Meropenem and Ciprofloxacin (5 cases each). Voriconazole was used in two admissions and Oseltamivir in one admission. Twelve cases required mechanical ventilation support. Overall, 7 patients died (2 renal and 5 cardiac transplant recipients). One patient died from a massive ischemic cerebral vascular accident. Four patients died due to septic shock: three patients harbored MDR *Pseudomonas aeruginosa*, and one patient harbored an ESBL producing *Escherichia coli*. One patient died from a cardiogenic shock. One patient died from a cerebral hemorrhage due to invasive aspergillosis. Serious infections leading to admission in the ICU or death was more common in the year following the transplantation (13/41 hospitalizations versus 17/117) (p = 0.03).

### Association between the studied variables and severity of infection

**Fig 4:**
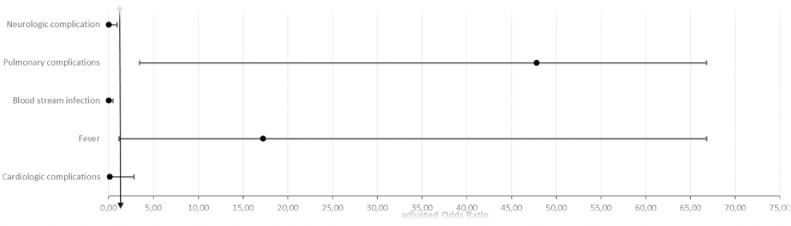
Regression plotting of risk factors for death.

In the study, bacteremia (aOR = 30.47), pulmonary complications (aOR = 65.96), neurologic complications (aOR = 44.29), and cardiac complications (aOR = 7.16) exhibited associations with a higher risk of mortality (Table 5) (Fig 4). It was noted that fever could have a protective effect against mortality resulting from infectious complications in our population (aOR = 0.05). Additionally, statistical analysis showed a correlation between acute kidney failure and hematologic complications with ICU admission as an outcome. However, no associations were identified between the type of infection, type of transplant, type of immunosuppressive drug, or comorbidities and the outcome of ICU admission or death.

**Table 5:** Univariate analysis of the association between the measured variables and death or ICU admission.

	Death		ICU admission	
Variables	OR (95% CI)	p-value	OR (95% CI)	p-value
Fever	0.075 (0.009–0.643)	**0.018**	1.275 (0.239–6.801)	0.776
Chills	0.000	0.997	1.712 (0.368–7.958)	0.493
Site of infection				
Respiratory tract	0.475 (0.089–2.527)	0.475	0.915 (0.198–4.321)	0.910
Urinary tract	1.430 (0.265–7.710)	0.677	1.430 (0.265–7.710)	0.677
Intra-abdominal	1.314 (0.149–11.579)	0.806	0.000	0.998
Skin and subcutaneous	0.000	0.999	2.630 (0.285–24.251)	0.394
Osteo-articular	0.000	0.999	4.028 (0.417–38.948)	0.229
Blood stream	5.912 (1.218–28.693)	**0.027**	0.000	0.998
Complications				
Renal	0.732 (0.085–6.326)	0.777	13.229 (2.424–72.189)	**0.003**
Neurologic	6.125 (0.591–63.485)	0.129	6.125 (0.591–63.485)	0.129
Metabolic	4.867 (0.489–48.397)	0.177	4.867 (0.489–48.397)	0.177
Hematologic	3.153 (0.567–17.532)	0.190	12.089 (2.467–59.227)	**0.002**
Digestive	4.633 (0.811–26.467)	0.085	4.633 (0.811–26.467)	0.085
Cardiovascular	7.150 (1.196–42.729)	**0.031**	2.630 (0.285–24.251)	0.394
Pulmonary	16.970 (3.365–85.567)	**0.001**	1.631 (0.183–14.533)	0.661
Type of transplantation	0.648 (0.200–2.105)	0.648	0.906 (0.304–2.706)	0.860
Immunosuppressant Drugs				
Prednisone	1.248 (0.144–10.808)	0.841	91196156.12 (0.000-NA)	0.998
Cyclosporine	1.137 (0.246–5.264)	0.869	1.137 (0.246–5.264)	0.869
Tacrolimus	0.992 (0.215–4.588)	0.992	0.515 (0.097–2.739)	0.437
MMF	0.329 (0.071–1.527)	0.156	0.329 (0.071–1.527)	0.156
Everolimus	1.884 (0.405–8.770)	0.420	1.884 (0.405–8.770)	0.420
Azathioprine	0.000	0.998	0.000	0.998
Sirolimus	0.000	0.998	1.231 (0.140–10.823)	0.851
Immunosuppressor overdosage	1.082 (0.707–1.654)	0.718	0.000	0.998
Isolated pathogen	0.405 (0.047–3.465)	0.409	0.000	0.998
Comorbidities				
HIV	0.000	0.999	0.000	0.999
Neoplasm	0.000	0.998	2.476 (0.451–13.600)	0.297
diabètes	0.915 (0.198–4.231)	0.910	0.475 (0.089–2.527)	0.383

Type of complications

Renal complications: acute renal failure, fluid electrolyte disorders, acute tubular necrosis.

Neurologic complications: peripheral neuropathy, convulsive seizures, ischemic stroke, Hemorrhagic Stroke.

Metabolic complications: gout arthritis, hyperglycemia, hypoglycemia.

Hematologic complications: anemia, leukopenia, thrombocytopenia, hematoma, Macrophage Activation Syndrome.

Digestive complications: gastritis, gastric hemorrhage, hepatic cytolysis, hepatic cholestasis.

Cardiovascular complications: acute myocardial infection, myocarditis, pericardial effusion, heart failure

Pulmonary complications: pulmonary edema, acute respiratory distress syndrome, pleural effusion, pulmonary emboli.

## Discussion

This study described the epidemiological, clinical, and microbiological characteristics of a transplant population admitted to the infectious diseases department at the Bichat Claude Bernard Hospital, Paris, France, between October 2017 and March 2019. It focused on different types of organ transplant in addition to wide spectrum of pathogens and infections in this specific population [24–26]. It highlighted the burden of infectious complications and associated mortality in transplant patients.

The demographic characteristics of the studied population were similar to other cohorts [7, 24] except for the prevalence of HIV infection and malignancy. In this study, 10 patients (9.6%) lived with HIV, and 19 patients (18.2%) had a malignant neoplasm. These findings might be explained by the fact that Bichat hospital is a specialized center for HIV and malignancy cases. HIV infection is no longer a contraindication for solid organ transplantation provided that HIV replication is fully suppressed, with excellent outcomes [27]. In comparison to this study, previous research included participants with a notably younger median age, particularly within the renal transplant patient subgroup [28–30]. This can be due to the earlier onset of end-stage renal disease in the majority of renal transplant candidates. This was not the case for other transplant types, in which patients had different profiles.

The median time from transplantation to infection was two years. Most patients were admitted several times including early and late after transplantation. This was also demonstrated in previous series [28, 31, 32]. In this study, we demonstrated that transplant patients who were admitted with early infections had a higher likelihood of having received immunosuppressive treatment with prednisone or mycophenolic acid. This finding aligns with expectations, as the increased incidence of infections in this patient population may be attributed to the potent immunosuppressive therapy received after transplantation. [33]. On the contrary, patients with multiple comorbidities, a history of neoplasm, or those receiving maintenance immunosuppressive therapy with everolimus experienced a higher incidence of infectious complications in the later stages following transplantation [34]. It is worth noting that at our center, the post-transplantation immunosuppressive regimen predominantly involves prednisone and mycophenolic acid in the majority of cases, with everolimus typically reserved for later stages after transplantation.

Bacterial infections, particularly pneumonia, urinary tract infections, and surgical site infections, were more prevalent than viral or fungal infections. They represented the predominant category of infectious complications following transplantation, particularly in the early post-transplantation period. These infections are associated with significant morbidity and mortality. This finding is consistent with the observations reported in both the Swiss transplant cohort [24] and the RESITRA cohort [35].

The predominant admission symptom within this cohort was fever, with approximately 66.5% of all admissions manifesting as febrile illnesses. This percentage was notably higher when compared to ICU admissions in the general population at the same center [36].

Respiratory tract infections were the most common cause of admissions (44%), followed by urinary tract infections (22%). This was expected since those are the most common infections seen in elderly and immunocompromised patients in France [37, 38]. There was no association of these infections with pulmonary transplantation potentially due to the limited sample size. Gram-negative bacilli infections, mainly urinary tract infections, respiratory, and sepsis, were the most common pathogens isolated in this retrospective cohort. Approximately 28.5% of patients exhibited clinical signs of infection without microbiological documentation. In such instances, the diagnosis of active infection primarily relied on imaging findings, assessment of inflammatory markers, and the response to empiric antibiotic therapy.

Thirteen patients contracted an infection from a multidrug-resistant gram-negative bacillus. Notably, this group had a high level of exposure to broad-spectrum antibiotics, immunosuppressive therapy, invasive medical procedures, and had undergone multiple hospitalizations both before and after transplantation [39–41]. There are currently no established standard recommendations for screening patients for bowel colonization with multidrug-resistant pathogens prior to transplantation nor for decolonization in patients with a history of drug-resistant bacterial infections [42].

Similarly to Bodro et al cohort [43], our descriptive findings also showed aspergillosis as the most common invasive fungal infection, while in the studies by Pappas et al. and Neofytos et al., candida ranked as the most prevalent [8, 34].

Thirty-one cases (20%) had a severe infection requiring ICU admission or leading to death, a percentage similar to other studies enrolling immunocompromised patients [44]. Infection stands as the leading cause of mortality in this population, with rates ranging from 21% to 63% among transplant patients during the first year following transplantation [9].

Mortality rates were elevated among patients who experienced bacteremia, pulmonary, neurologic, or cardiac complications, as well as those requiring ICU admissions. These findings likely stemmed from the fact that these patients often developed septic shock accompanied by complications related to the infection [45]. However, it’s essential to exercise caution when interpreting the associations between major variables and mortality due to the limited number of patients with the outcome.

Despite its contribution in shedding light on the frequency, risk factors, treatment, and outcomes of infections in a tertiary center in Paris within the transplant population, this study has several limitations. It was conducted as a retrospective analysis using clinical chart data, which may introduce misclassification bias and result in missing or unknown data. Additionally, during the study period, not all transplant patients with infections were admitted to our department, particularly those who had undergone lung and liver transplants, as they were typically managed in their respective departments even when seeking treatment for infectious complications. The study’s sample size did not permit specific infection and outcome analyses, and it did not provide insights into long-term complications or post-transplant prophylaxis treatments.

In conclusion, this study underscores the substantial burden of infections in solid organ transplant recipients and emphasizes the association between infectious complications, high morbidity, and severe immunosuppression. Therefore, transplant patients with these identified risk factors necessitate thorough evaluation, extended antimicrobial prophylaxis, and a judicious approach to empiric treatment tailored to the severity of the infection.

## Supporting information

S1 Database (XLSX)
